# Injectable Hydrogel Combined with Nucleus Pulposus-Derived Mesenchymal Stem Cells for the Treatment of Degenerative Intervertebral Disc in Rats

**DOI:** 10.1155/2019/8496025

**Published:** 2019-10-15

**Authors:** Feng Wang, Li-ping Nan, Shi-feng Zhou, Yang Liu, Ze-yu Wang, Jing-cheng Wang, Xin-min Feng, Liang Zhang

**Affiliations:** ^1^Department of Orthopedics, Dalian Medical University, Dalian, 116000 Liaoning, China; ^2^Department of Orthopedics, Clinical Medical College of Yangzhou University, Yangzhou, 225001 Jiangsu, China

## Abstract

Stem cell-based tissue engineering in treating intervertebral disc (IVD) degeneration is promising. An appropriate cell scaffold can maintain the viability and function of transplanted cells. Injectable hydrogel has the potential to be an appropriate cell scaffold as it can mimic the condition of the natural extracellular matrix (ECM) of nucleus pulposus (NP) and provide binding sites for cells. This study was aimed at investigating the effect of injectable hydrogel-loaded NP-derived mesenchymal stem cells (NPMSC) for the treatment of IVD degeneration (IDD) in rats. In this study, we selected injectable 3D-RGD peptide-modified polysaccharide hydrogel as a cell transplantation scaffold. In vitro, the biocompatibility, microstructure, and induced differentiation effect on NPMSC of the hydrogel were studied. In vivo, the regenerative effect of hydrogel-loaded NPMSC on degenerated NP in a rat model was evaluated. The results showed that NPMSC was biocompatible and able to induce differentiation in hydrogel in vivo. The disc height index (almost 87%) and MRI index (3313.83 ± 227.79) of the hydrogel-loaded NPMSC group were significantly higher than those of other groups at 8 weeks after injection. Histological staining and immunofluorescence showed that the hydrogel-loaded NPMSC also partly restored the structure and ECM content of degenerated NP after 8 weeks. Moreover, the hydrogel could support long-term NPMSC survival and decrease cell apoptosis rate of the rat IVD. In conclusion, injectable hydrogel-loaded NPMSC transplantation can delay the level of IDD and promote the regeneration of the degenerative IVD in the rat model.

## 1. Introduction

Lower back pain (LBP) is a common disease with high incidence [1] and imposes an enormous economic burden on the society and family [2]. The prevalence of LBP is increasing due to the aging of population [3], and intervertebral disc degeneration (IDD) is considered associated with LBP [4]. Unfortunately, none of the common therapy can effectively repair or regenerate the structure and function of the degenerative intervertebral disc (IVD) [5, 6]. Thus, it is necessary to develop a new approach for IDD.

IVD is composed of three parts: the central gelatinous NP, the outer multilaminate annulus fibrosus (AF), and the cartilage endplate [7]. NP is one of the most critical parts of IVD, which can provide a suitable extracellular environment for the growth and secretion function of NP cells [8]. Therefore, it is believed that restoration of a degenerated NP may be of great importance to the treatment of IDD. The function and numbers of the endogenous IVD cells are decreasing during the IDD procedure, which results in failure of cell-based endogenous repair [9]. Mesenchymal stem cell- (MSC-) based therapy has broad application prospects for the treatment of IDD. Bone marrow-derived MSC [10], adipose-derived MSC [11], human umbilical cord-derived MSC [12], and other types of stem cells [13] have been used to treat IDD. Unfortunately, the local microenvironment of the degenerative IVD, which is characterized by hypertonicity, acidic pH, limited nutrition, and oxygen [2, 14, 15], impaired cell viability, cell proliferation ability, and biosynthesis ability of ECM [16, 17]. In 2007, Risbud et al. [18] confirmed the existence of endogenous progenitor cells in human NP. Other studies further confirmed that these kinds of endogenous NP-derived MSC (NPMSC) show a stronger tolerance to the harsh microenvironment compared with other types of MSC [19, 20]. NPMSC have got increasing attention and show remarkable prospects for the regeneration of degenerative IVD [21].

**Figure 1:**
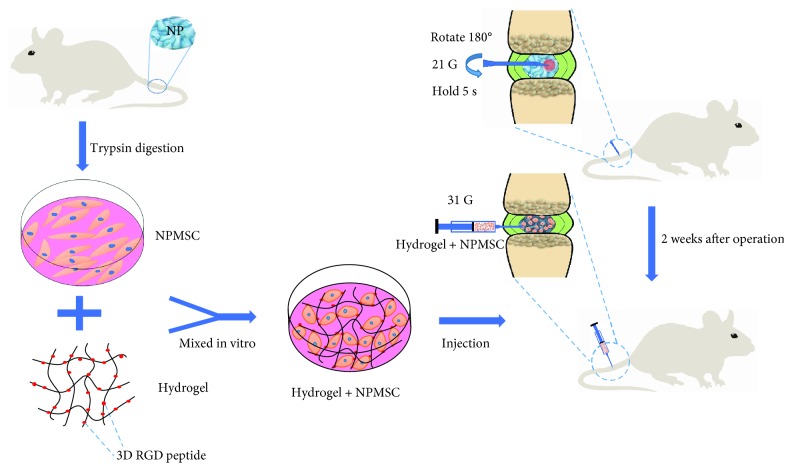
Schematic of the basic process of the present study. NPMSC were isolated from the coccygeal IVD of SD rats, and amplification was performed in vitro. After IDD model induction by a 21G needle, injectable hydrogel-loaded NPMSC was transplanted into the degenerated NP by a microsyringe.

The survival of transplanted MSC is a major obstacle for MSC transplantation therapy [2]. MSC transplantation alone cannot improve the local adverse microenvironment [15]. The scaffold-loaded cell transplantation can not only transplant cells into the target locations but also create a suitable microenvironment for the better survival of transplanted MSC [22]. Due to the rheological and mechanical properties of hydrogel similar to those of the native NP [23, 24], injectable hydrogel has become the preferred material for NP repair. In this study, we aimed to investigate the regenerative effects of injectable hydrogel combined with NPMSC in a rat model of IDD. A schematic outline of the study is depicted in Figure 1.

## 2. Materials and Methods

### 2.1. Animal Care and Use

Seventy healthy Sprague-Dawley (SD) rats (weight, 180-220 g; age, 3-4 months) were provided by the Laboratory Animal Center of Jiangsu University (License no. SCXK (Su) 2018-0012). Animal care and use followed the guidelines of Laboratory Animals published by the US National Institutes of Health. All experiments were approved by the Institutional Animal Care and Use Committee of Yangzhou University. Animals were bred in a rat box at a controlled environment (26 ± 3°C, 12 : 12 h light/dark) with a relative humidity of 70-85%. Experimental animals were not restricted to get water and standard diet.

### 2.2. Isolation and Culture of NPMSC

Primary NPMSC were isolated from the coccygeal IVD tissues of twenty SD rats. The rats were killed by an overdose of pentobarbital, and their tails were taken. Coccygeal IVD were harvested under aseptic and light microscope conditions. The gel-like NP tissue sample was washed three times with phosphate-buffered saline (PBS) containing 1% penicillin-streptomycin (Gibco, MD, USA) and digested with 0.2% collagenase type II (Gibco, MD, USA) solution at 37°C for 2 h. After centrifugation at 800 g for 5 min, the cell pellets were cultured in MSC culture medium (Cyagen Biosciences, Guangzhou, China) supplemented with 20% fetal bovine serum (HyClone, UT, USA) and 1% penicillin/streptomycin at 37°C with 5% CO_2_ of humidified atmosphere. The medium with suspension cells was removed after 1 day; then, the medium was completely changed twice a week. The cells were passaged at a 1 : 3 ratio at 80-90% confluence. After three passages, the NPMSC were used.

### 2.3. Immunophenotypic Characterization

In accordance with the appraisal standards of stem cells proposed by the International Society for Cellular Therapy (ISCT), the NP-progenitor cell surface marker Tie2 and the MSC surface markers CD73, CD90, CD105, CD34, and CD45 were measured. Isotype controls (BD Biosciences, NJ, USA) were used in each case as the negative control. To put it simply, 2 × 10^5^ NPMSC were incubated with monoclonal primary antibodies (CD105-phycoerythrin (PE), CD90-fluorescein isothiocyanate (FITC), CD73-PE, CD45-FITC, CD34-PE, and Tie2-FITC (BD Biosciences, NJ, USA)) for 30 min at room temperature in the dark. Then, cells were washed twice with cold PBS and resuspended in 500 *μ*L PBS to produce a single cell solution, and specific surface marker expression was detected using flow cytometry (FACSCalibur, BD Biosciences).

### 2.4. Multipotent Differentiation

The multilineage differentiation potentials of NPMSC were characterized by osteogenic, adipogenic, and chondrogenic differentiation. Briefly, NPMSC were resuspended at a density of 5 × 10^5^/mL and seeded in 6-well plates. When cultured cells reached 80% confluence, the cells were cultured with osteogenic, adipogenic, and chondrogenic differentiation kits (Cyagen Biosciences, Guangzhou, China). According to the manufacturer's instructions, after washing with PBS and fixing with 4% paraformaldehyde for 20 min, Alizarin red, Oil Red O, and Alcian blue were used when the differentiation reached the required days. Stained areas were observed under a fluorescence microscope (Leica, Wetzlar, Germany).

### 2.5. The Optimum Concentration of Hydrogels

VitroGel 3D-RGD hydrogel was purchased from TheWell Bioscience (NJ, USA). Cell Counting Kit-8 (CCK-8, Dojindo, Japan) and lactate dehydrogenase (LDH) assay kit (Beyotime Biotechnology, Haimen, China) were used to detect the optimum concentration of the hydrogel. The hydrogel solution was mixed directly with Hank's (HyClone, UT, USA) at 1 : 0, 1 : 1, 1 : 2, and 1 : 3 (hydrogel : Hank's, *v*/*v*) ratios at room temperature. NPMSC were resuspended at a density of 5 × 10^5^/mL and mixed with different concentrations of hydrogels at the ratio of 1 : 4. Hydrogel-loaded NPMSC were cultured for 3, 7, and 14 days before determination. At each time point, CCK-8 solution (10% CCK-8 in fresh DMEM) was added and incubated at 37°C under 5% CO_2_ for 3 h. The supernatant (100 *μ*L) was collected and measured for the absorbance values at 450 nm. The cytotoxicity of different concentrations of hydrogel on NPMSC was detected using the LDH assay kit. The supernatant was collected at 3, 7, and 14 days and tested for the absorbance values at 450 nm by a microplate reader (Bio-Rad, Hercules, CA, USA).

### 2.6. Microstructure

As referred to the results above, the hydrogel was mixed with Hank's in the optimal concentration of hydrogel ratio; then, liquid medium was mingled with diluted hydrogels at the ratio of 1 : 4 at 37°C for 30 min. The specimens were lyophilized under vacuum and cut into small pieces with a thickness of 1 mm. Then, the samples were coated with gold and analyzed by a scanning electron microscope (SEM) at an accelerating voltage of 5 kV (SUPPA 55, ZEISS, Germany).

### 2.7. Cell Proliferation

For cell proliferation, 4′,6-diamidino-2-phenylindole (DAPI) and 5-ethynyl-2′-deoxyuridine (Edu) (Beyotime, Haimen, China) staining was used in in vitro 3D culture (hydrogel group) or 2D well plate culture (control group). Briefly, the cell suspension and hydrogel-loaded NPMSC were inoculated in 96-well plates (100 *μ*L/well). After 1, 4, and 8 days after culture, cells were fixed with 4% paraformaldehyde for 10 min and washed, and then, cells were incubated with 100 *μ*L DAPI at ambient temperature for 5 min. In addition, at 8 days after culture, cells of the two groups were incubated with Edu working solution as described by the manufacturer's protocol after being digested or released. The stained samples were photographed under a fluorescence microscope (Olympus Europe, Hamburg, Germany). ImageJ software (Wayne Rasband, National Institute of Health, USA) was used for image analysis. Three randomly selected fields from three samples were utilized for calculation.

### 2.8. Western Blot

NPMSC mixed with optimum concentration of hydrogel and inoculated (2.4 mL/well) in a 6-well plate was considered a hydrogel group, and NPMSC suspension cultured in the 6-well plate was considered a control group. Both groups were cultured in MSC differentiation kits (Cyagen Biosciences, Guangzhou, China) for 2 weeks. When the scheduled time reached, the cells were obtained and the total protein were extracted in RIPA buffer containing 1% PMSF on ice for 30 min. Protein supernatant was obtained by centrifugation for 20 min at 4°C, and protein concentration was measured using a bicinchoninic acid protein assay kit (Beyotime Institute of Biotechnology, Haimen, China). Following electrophoresis in 10% sodium dodecyl sulfate-polyacrylamide gel, the separated proteins were transferred onto polyvinylidene fluoride membranes (EMD Millipore, MA, USA). The membranes were blocked with 5% nonfat milk for 2 h at room temperature, then incubated with primary antibodies 12 h at 4°C. The primary antibodies were as follows: collagen type II (1 : 5000; Abcam, Cambridge, UK), aggrecan (1 : 1000; Abcam), and *β*-actin (1 : 10000; ABclonal, Wuhan, China). After washing three times with Tris-buffered saline and 0.1% Tween 20, the membranes were incubated with horseradish peroxidase- (HRP-) labeled secondary antibodies (Sanying, Wuhan, China) for 2 h on a shaker at room temperature. Bands were visualized using an enhanced chemiluminescence system. The relative amount of protein was analyzed using ImageJ software. *β*-Actin was used as the loading control.

### 2.9. IDD Model Induction and NPMSC Implantation

An SD rat IDD model was established according to the method reported in our previous study [25]. Briefly, the rats were anesthetized using intraperitoneal injection of 50-80 mg/kg pentobarbital. After sterilization with povidone iodine, coccygeal (Co) 5-6, Co6-7, Co7-8, and Co8-9 were percutaneously punctured by a 21G needle to a depth of 5 mm. Two weeks later, those different levels were divided into four groups: hydrogel+cell group (Co5-6, hydrogel-loaded NPMSC injection), hydrogel group (Co6-7, hydrogel injection only), PBS group (Co7-8, PBS injection only), and cell group (Co8-9, NPMSC injection only), while the Co4-5 level was designated as the control group.

To visualize the survival situation of transplanted NPMSC, the injected NPMSC expressing mCherry were generated. Following the manufacturer's protocol, when 50% confluence was reached, the cells were transfected with GV326 lentivirus vector-mCherry (GeneChem, Shanghai, China) at a multiplicity of infection (MOI) of 10, 20, 50, or 100. The fluorescence microscope was used to detect the expression of mCherry fluorescent protein 72 h later. Considering the transfection efficiency and cell damage, MOI = 100 was selected for succeeding infection. The NPMSC were transferred to a complete medium after 18 h of transfection. After 2 g/mL puromycin (Sigma-Aldrich, MO, USA) screening, the stably transfected NPMSC were used for transplantation. The 31G needle of a microsyringe was carefully inserted in the middle of the disc, and the volume of injection of each segment was 2 *μ*L. The injection process was delayed for approximately 5 min to minimize leakage [26].

### 2.10. In Vivo Tracking

At 1 day and 30 days after injection, rats were anesthetized with the method as described above. After precooling the camera, anesthetized rats were sequentially placed in the in vivo imaging system (IVIS) chamber and the tail was placed in the center of panel. The IVIS Lumina III (Xenogen, CA, USA) was used to detect the signal intensity of mCherry fluorescent protein in the injected level (excitation wavelength: 587 nm; emission wavelength: 610 nm). The fluorescent signals were overlaid on a grayscale of the rat and quantified as mean total radiant efficiency ([photons/s]/[mW/cm^2^]) ± s.e.m. in a region of interest (ROI) via the Living Image software (PerkinElmer). The assured ROI was labeled at Co5-6 and Co8-9 where the NPMSC is transplanted. The total luminescence within ROI was procured from the overlaying luminescent images in the IVIS directory.

### 2.11. Radiographic Analysis

To assess the disc height, lateral radiographs of the coccygeal discs were taken preoperation, preinjection, and 1, 2, 4, and 8 weeks after injection under anesthesia as described above. Two independent imaging technologists who were blinded to the study design assessed the disc height index (DHI). According to the method of Han et al. [27], DHI% was calculated using the following formula: DHI% = (postpunctured DHI/prepunctured DHI) × 100%. The change of DHI expressed as DHI% (postinjection DHI/preinjection DHI) was used to describe disc height change.

MRI (3.0T, GE, CT, and US) taken preoperation, preinjection, and 1, 2, 4, and 8 weeks after injection was used to evaluate the signal and structural change. Shortly, the rats were anesthetized by intraperitoneal injection of 50-80 mg/kg pentobarbital and maintained by inhalation of 2% anesthetic isoflurane. Sagittal T2-weighted images were obtained in the following settings: (a) fast spin echo sequence with a time to repetition (TR) of 3000 ms and time to echo (TE) of 45 ms, (b) 9 excitations and 5 cm field of view, and (c) segment thickness of 0.5 mm with a 0 mm gap. The MRI images were evaluated by two radiologists who were blinded to the study design using the MRI index to evaluate the rehydration of NP tissue [28].

### 2.12. Histological Analysis

After MRI examination, the rats were killed by an intraperitoneal overdosage injection of pentobarbital and the tails were harvested at 4 weeks and 8 weeks after injection. The specimens were fixed with formaldehyde, decalcified with 10% ethylenediaminetetraacetic acid solution, and embedded in paraffin. Sections were stained with HE, safranin O (SO), and toluidine blue staining and then captured under a microscope. The degeneration score was evaluated by two independent observers as previously reported [11, 28].

### 2.13. Macroscopic Observation of IVD

At 8 weeks after injection, the rats were killed as described above and tails were obtained. After being washed with PBS, the surgical blade was used to separate the adjacent vertebrae along the middle of the disc under fluoroscopic guidance. The NP tissue was photographed, and the area in the cross section was analyzed by ImageJ software, which roughly represents the repair and regeneration of NP.

### 2.14. Terminal Deoxynucleotidyl Transferase-Mediated dUTP Nick End Labeling (TUNEL) Assay

IVD sections were obtained by the above method at 3 days after operation and 4 weeks and 8 weeks after injection; apoptosis-related DNA damage was evaluated by TUNEL staining. In brief, after being fixed in 4% paraformaldehyde solution for 30 min at 37°C, permeabilized in a solution containing 0.5% Triton X-100 for 5 min, and washed twice with PBS, sections were stained with freshly prepared TUNEL working liquid (Beyotime, Haimen, China) at room temperature for 1 h in the dark. Afterward, the sections were washed and stained with Fluorochrome DAPI (Beyotime, Haimen, China) for 10 min. The results were photographed by a fluorescence microscope and analyzed by ImageJ software.

### 2.15. Immunofluorescent Staining

The rats were killed as described above and the tails were obtained. The specimens of the whole IVD with adjacent vertebral bodies were harvested and cut into 5 *μ*m with a freezing microtome (Leica, Wetzlar, Germany) at 8 weeks after injection. After being fixed in 4% paraformaldehyde for 30 min, washed twice with PBS, and blocked in 5% bull serum albumin for 1 h, the tissue sections were incubated, respectively, overnight with primary antibodies at 4°C: rabbit polyclonal anti-collagen type II and anti-aggrecan (1 : 100, Abcam, Cambridge, UK). After washing with PBS, slides were incubated, respectively, with FITC-conjugated and Cy3-conjugated goat anti-rabbit IgG (1 : 10000, ABclonal, Wuhan, China) secondary antibodies for 2 h at room temperature in the dark. The immunostaining results were photographed by a fluorescence microscope and analyzed by Image-Pro Plus 6.0 software.

### 2.16. Real-Time Polymerase Chain Reaction (RT-PCR)

Quantification of collagen type II and aggrecan mRNA levels was conducted using RT-PCR at 8 weeks after injection. Total RNA was extracted using a TRIzol reagent (Invitrogen, Carlsbad, CA, USA). According to the instructions, the PrimeScript RT reagent kit and SYBR Premix Ex Taq (Vazyme Biotech, Nanjing, China) were used to transcribe reversely RNA to cDNA and amplify the cDNA. The mRNA expression levels of collagen type II and aggrecan were calculated by the comparative Ct method. Primer sequences are listed in Table 1.

### 2.17. Statistical Analysis

The quantitative data were expressed as the mean ± standard deviation (SD). The SPSS software (version 19.0 for Windows, SPSS, IL, US) was used to conduct statistical analysis. The data of multiple independent groups was analyzed by one-way ANOVA. The LSD *t*-test was used to analyze the data of two group parameters. When the *p* value is less than 0.05, the difference was considered significant.

## 3. Result

### 3.1. Evaluation of Isolated Cells

**Figure 2:**
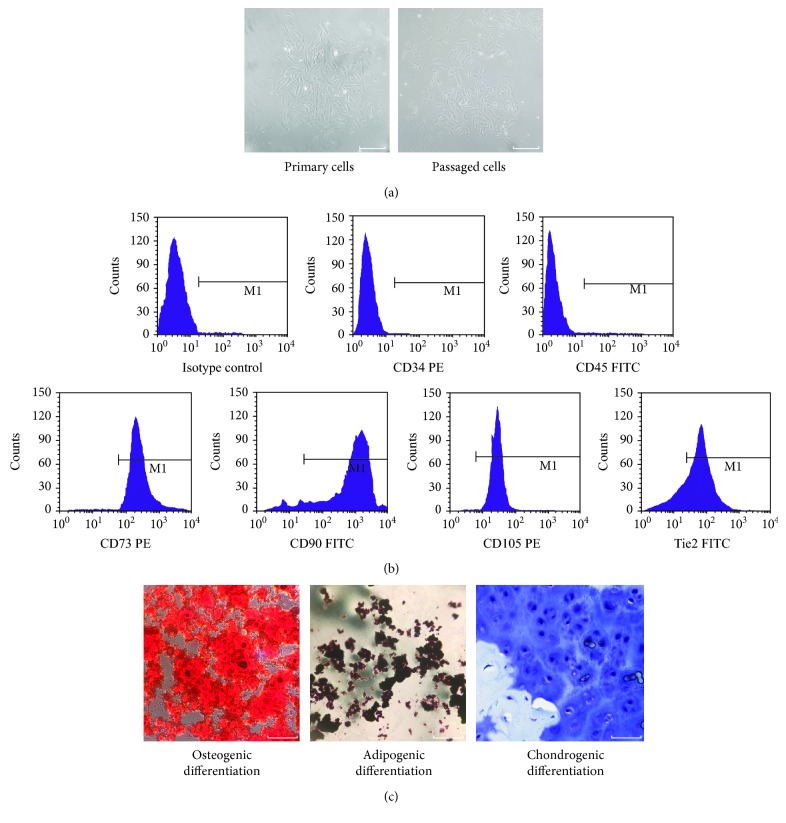
Identification of nucleus pulposus-derived mesenchymal stem cells (NPMSC). (a) Isolated primary cells presented with a long spindle shape and showed colony growth; the passaged cells were spindle-shaped. (b) NPMSC had higher expressions (>98%) of MSC surface markers CD73, CD90, and CD105 and lower expressions (<2%) of hematopoietic stem cell surface markers CD34 and CD45 and higher expressions (>80%) of Tie2. (c) NPMSC was positive for Alizarin red, Oil Red O, and Alcian blue staining after induced differentiation. White scale bar = 50 *μ*m.

Cells isolated from rat coccygeal IVD tissues presented with a long spindle shape and showed colony growth, indicating the ability to adhere to plastic (Figure 2(a)). As shown in Figure 2(b), flow cytometry identified that the cells highly expressed CD105 (98.22%), CD90 (97.54%), and CD73 (97.55%) which are usually positive in MSC and inferiorly expressed CD34 (1.42%) and CD45 (0.95%) which are negative in MSC, while the cells highly expressed Tie2 (81.76%) which is positive in NP-progenitor cells. The multilineage differentiation was certified by induction into the osteogenic, chondrogenic, and adipogenic lineages in vitro (Figure 2(c)). All results showed that the cells corresponded to the evaluation criteria of MSC described by the ISCT.

### 3.2. Proper Concentration of the Hydrogel and Microstructure of the Hydrogel

**Figure 3:**
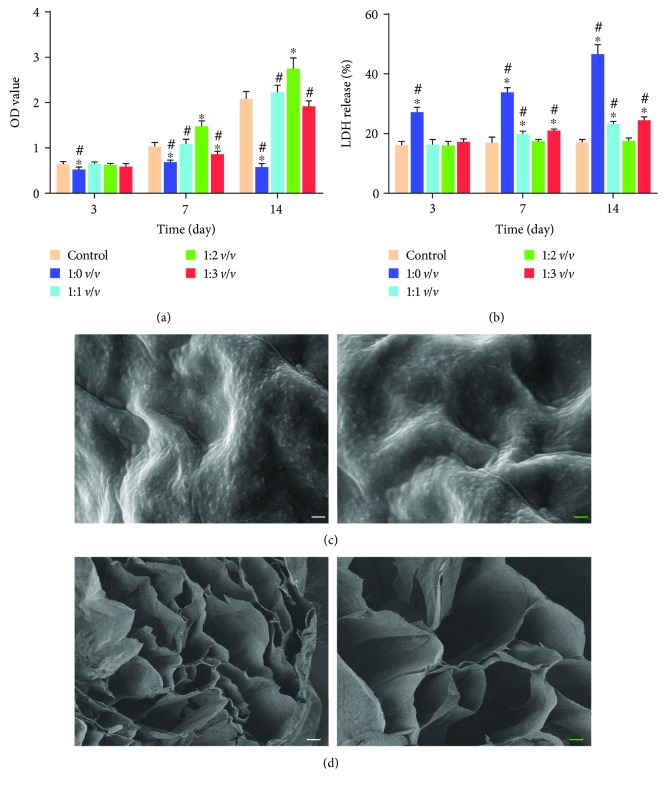
The proper concentration of hydrogel and microstructure of the hydrogel. (a) The proliferation of NPMSC cultured in different concentrations of hydrogels. (b) The cytotoxicity of different concentrations of hydrogel for NPMSC. (c) SEM image of the 1 : 2 *v*/*v* hydrogel surface. (d) Cross sections of 1 : 2 *v*/*v* hydrogel. Data represent mean ± SD; the error bars represent the standard deviation of measurements for 6 separate samples (*n* = 6); ^∗^*p* < 0.05 compared with control group; ^#^*p* < 0.05 compared with 1 : 2 *v*/*v* group. White scale bar = 100 *μ*m, blue scale bar = 50 *μ*m.

As shown in Figure 3(a), the cell viability of all other groups was not significantly different from the control group except the 1 : 0 *v*/*v* group at day 3. Nonetheless, the cell viability of the 1 : 2 *v*/*v* group was significantly higher than those of other groups at days 7 and 14. In addition, the released LDH reflected the cytotoxicity of hydrogel. The LDH of the 1 : 0 *v*/*v* group was higher than those of the other groups, and the difference was statistically significant. The LDH of the 1 : 1 *v*/*v* group and 1 : 3 *v*/*v* group were higher than that of the control group at day 14. But there was no significant difference between the 1 : 2 *v*/*v* group and the control group at each time point of LDH (Figure 3(b)). As shown in Figures 3(c) and 3(d), the hydrogel at the concentration of 1 : 2 *v*/*v* displayed an irregular porous structure and the pores were well connected which would facilitate cell migration. Hence, the optimum concentration of hydrogel for NPMSC was identified as 1 : 2 *v*/*v*.

### 3.3. Cell Proliferation and Differentiation

**Figure 4:**
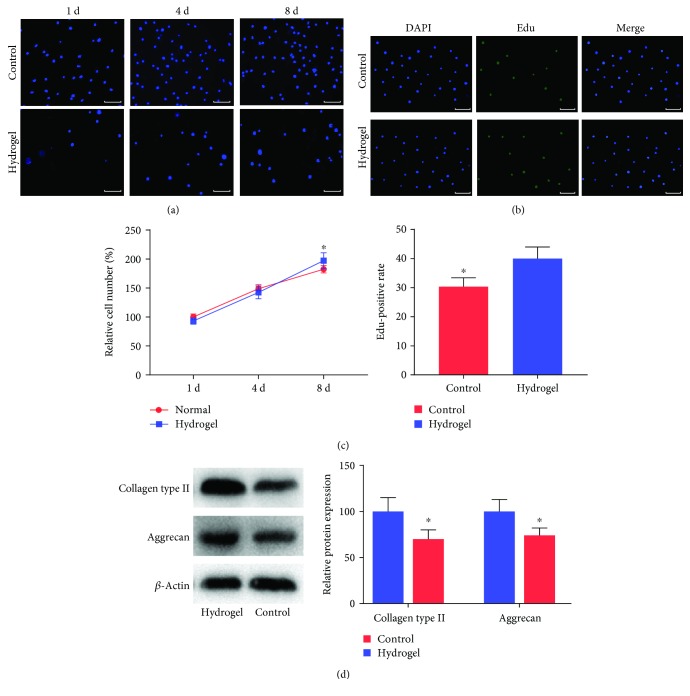
Cell proliferation and differentiation. (a) Representative images of DAPI staining for 2D (control group) and 3D (hydrogel group) cultured NPMSC at 1, 4, and 8 days after cultivation. (b) Representative images of Edu staining for 2D (control group) and 3D (hydrogel group) cultured NPMSC at 8 days after cultivation. (c) Investigation of growth rate and Edu-positive rate of NPMSC culture for 8 days in the control group and hydrogel group. (d) Relative protein levels of collagen type II and aggrecan in the control group and hydrogel group. Data represent mean ± SD; the error bars represent the standard deviation of measurements for 3 separate samples (*n* = 3); ^∗^*p* < 0.05 compared with the hydrogel group. White scale bar = 50 *μ*m.

The proliferation of NPMSC cultured in the well plate and hydrogel was determined by cell number counting and the Edu method. The cell number of both groups increased gradually at each time point (Figure 4(a)). There was no significant difference between the two groups in terms of cell proliferation rate at day 4; however, the cell growth rate of the hydrogel group was significantly higher than that of the control group at day 8 (Figure 4(c)). At 8 days after culture, the rate of Edu-positive cells in the hydrogel group was significantly higher than that of the control group (Figures 4(b) and 4(c)). Collagen type II and aggrecan are specific extracellular matrices (ECM) of NP cells. The relative protein expressions of collagen type II and aggrecan in the hydrogel group were significantly higher than those of the control group (Figure 4(d)).

### 3.4. Cell Survival in the Transplanted IVD

**Figure 5:**
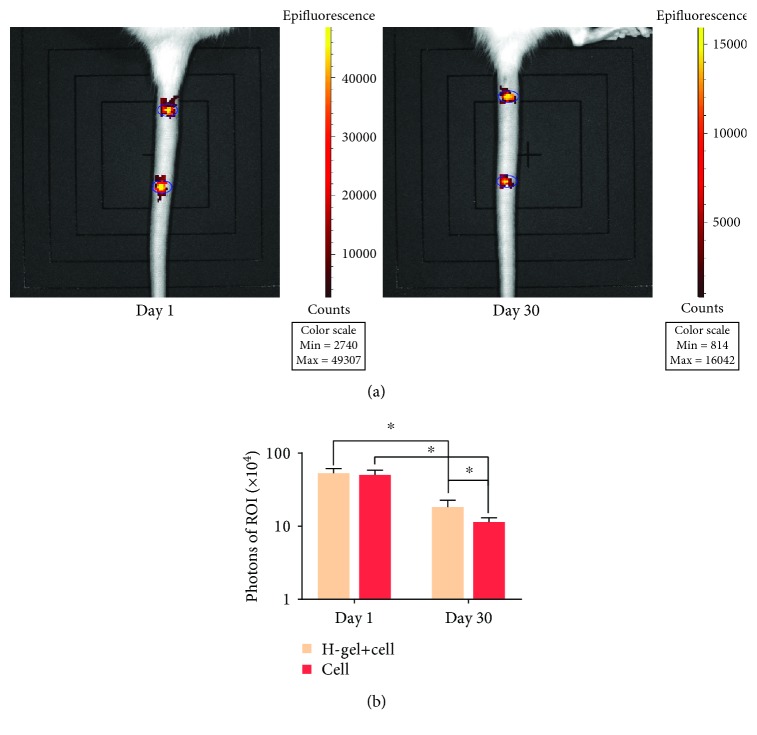
Cell retention in the IVD. (a) Representative images of IVIS at day 1 and day 30 after injection. (b) The histogram of luminescence of the hydrogel+cell group and cell group in the ROI in overlaying images. ROI was represented by a blue circle. The H-gel+cell group is the hydrogel+cell group. Data represent mean ± SD; the error bars represent the standard deviation of measurements for 3 separate samples (*n* = 3); ^∗^*p* < 0.05 compared with the hydrogel+cell group.

To visualize the survival situation of transplanted NPMSC in vivo, the IVIS system was used to detect the luminescence of ROI after 1 day and 30 days after injection. As shown in Figures 5(a) and 5(b), there was no significant difference in the luminescence of defined ROI between the hydrogel+cell group and the cell group at 1 day after injection. The photons of both groups decreased significantly with time, and the luminescence of ROI in the hydrogel+cell group was significantly stronger than that of the cell group at 30 days after injection. Thus, it was indicated that hydrogel can be used as a carrier to support the long-term survival of NPMSC in vivo.

### 3.5. Radiographic and MRI Evaluation

**Figure 6:**
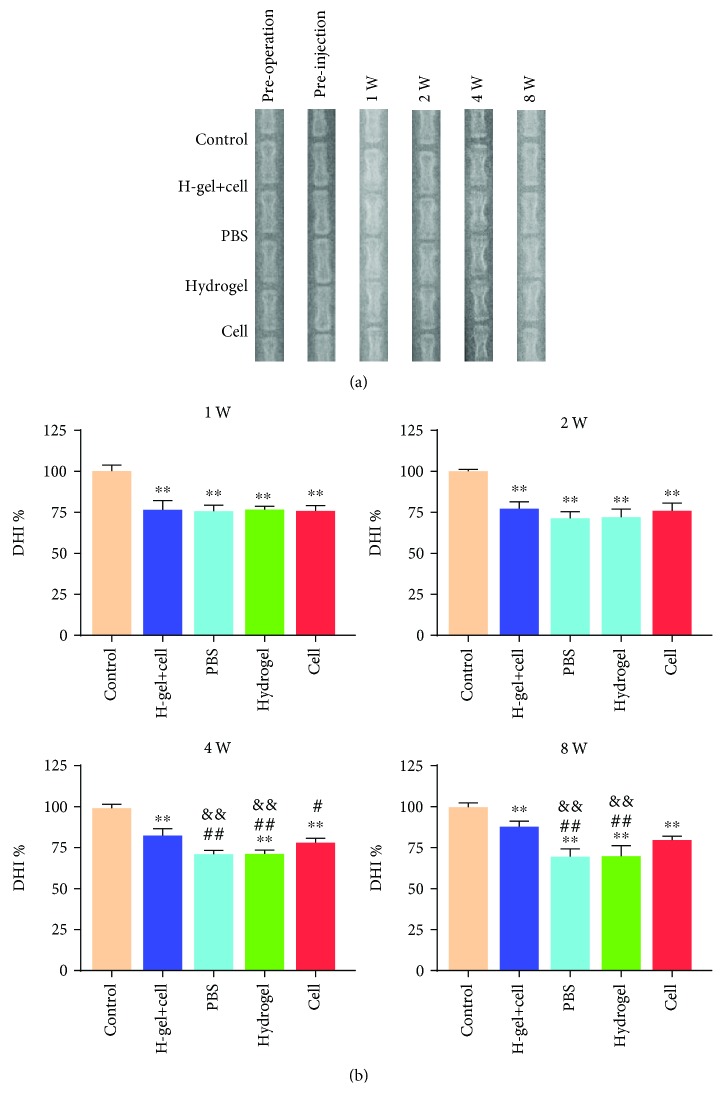
Typical radiographs and DHI% of different groups preoperation, preinjection, and 1, 2, 4, and 8 weeks after injection. (a) The radiographs of different groups. (b) DHI% was applied to quantitatively evaluate the changes of disc height. The H-gel+cell group is the hydrogel+cell group. All data are expressed as the mean ± SD; the error bars represent the standard deviation of measurements for 6 separate samples (*n* = 6); ^∗^*p* < 0.05, ^∗∗^*p* < 0.01 compared with control group; ^#^*p* < 0.05, ^##^*p* < 0.01 compared with hydrogel+cell group; ^&^*p* < 0.05, ^&&^*p* < 0.01 compared with cell group.

To explore the degree of IDD after injection, the DHI% was calculated. As shown in Figures 6(a) and 6(b), the DHI% remained stable in the control group at each time point. The DHI% in all other groups were significantly lower than that of the control group after injection. There was no significant difference among different groups at 1 week and 2 weeks after injection. The DHI% of the hydrogel+cell and cell groups were significantly higher than those of the PBS group and hydrogel group at 4 weeks and 8 weeks after injection. In addition, there was no significant difference between the hydrogel+cell group and cell group at 2 weeks and 4 weeks after injection, but a significant difference at 8 weeks.

**Figure 7:**
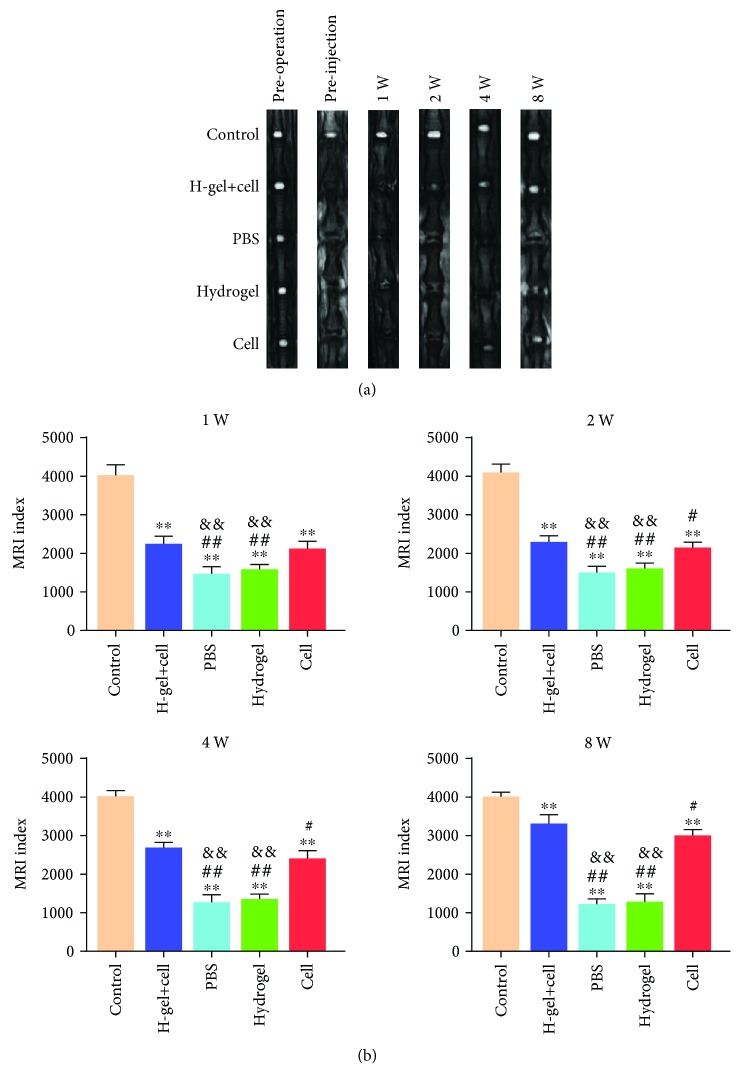
MRI images and MRI index of different groups. (a) Typical T2-weighted MRI images of different groups preoperation, preinjection, and 1, 2, 4, and 8 weeks after injection. (b) The MRI index was applied to quantitatively evaluate the rehydration and regeneration of degenerative disc. The H-gel+cell group is the hydrogel+cell group. All data are expressed as the mean ± SD; the error bars represent the standard deviation of measurements for 6 separate samples (*n* = 6); ^∗^*p* < 0.05, ^∗∗^*p* < 0.01 compared with control group; ^#^*p* < 0.05, ^##^*p* < 0.01 compared with hydrogel+cell group; ^&^*p* < 0.05, ^&&^*p* < 0.01 compared with cell group.

The MRI was used to evaluate the signal and structural change of NP tissue (Figures 7(a) and 7(b)). The MRI indexes of the PBS group and hydrogel group were lower than those of the hydrogel+cell group and cell group at 2, 4, and 8 weeks after injection. Additionally, there was no significant difference between the hydrogel+cell group and the cell group at 1 and 2 weeks after injection, but a significant difference at 4 and 8 weeks after injection.

### 3.6. Histological Analysis and Macroscopic Observation of IVD

**Figure 8:**
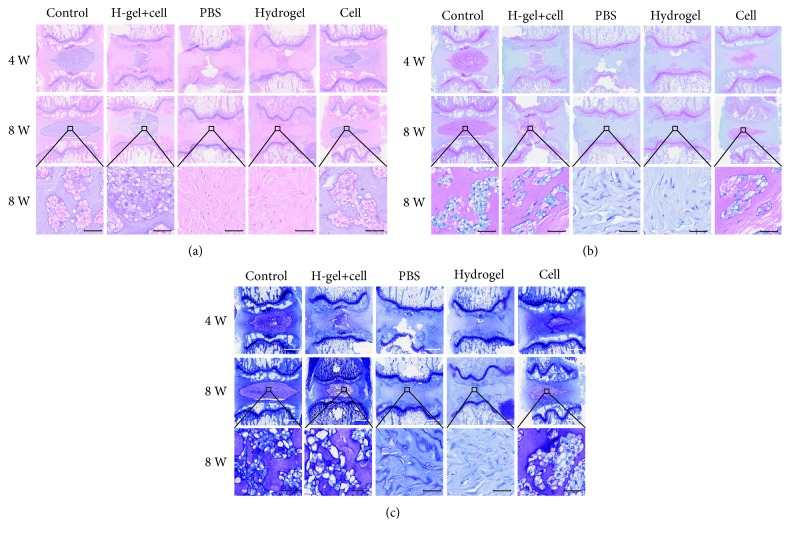
HE, S-O, and toluidine blue staining of different groups. (a) Representative HE staining at 4 and 8 weeks after injection. (b) Representative S-O staining at 4 and 8 weeks after injection. (c) Representative toluidine blue staining at 4 and 8 weeks after injection. The H-gel+cell group is the hydrogel+cell group. White scale bar = 1 mm, black scale bar = 50 *μ*m.

**Figure 9:**
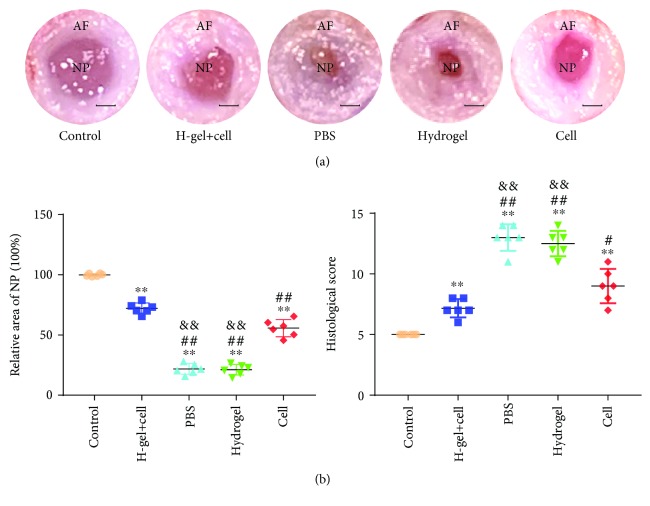
Macroscopic observation and histological score of different groups. (a) Macroscopic images of different groups at 8 weeks after injection. (b) The relative area of nucleus pulposus at 8 weeks after injection. (c) Histological scores obtained at 8 weeks after injection. The H-gel+cell group is the hydrogel+cell group. Data represent mean ± SD; the error bars represent the standard deviation of measurements for 6 separate samples (*n* = 6); ^∗^*p* < 0.05, ^∗∗^*p* < 0.01 compared with control group; ^#^*p* < 0.05, ^##^*p* < 0.01 compared with hydrogel+cell group; ^&^*p* < 0.05, ^&&^*p* < 0.01 compared with cell group. Scale bar = 1 mm.

HE staining (Figure 8(a)) showed that NP tissue was well organized and homogeneous in the control group at each point after injection. In the contrary, NP tissue was destroyed or even disappeared, and AF tissue was disorganized and lost its concentric lamellar structure in the PBS group and hydrogel group. The NP tissue almost disappeared and was filled with fibrillar connective tissues in the PBS group and hydrogel group at 8 weeks after injection. The structure of NP and ECM distributions was clearly visible in the hydrogel+cell group and cell group, but the quantity and structure of the ECM in the hydrogel+cell group were much better than those of the cell group. S-O staining (Figure 8(b)) showed that the proteoglycan levels decreased significantly in the PBS group and hydrogel group, but the hydrogel+cell group and cell group exhibited stronger staining than the PBS group and hydrogel group. Additionally, the hydrogel+cell group exhibited stronger staining than the cell group. Toluidine blue staining revealed that the hydrogel+cell group and cell group exhibited more NP chondrocytes and positive proteoglycan staining than the PBS group and hydrogel group (Figure 8(c)). In addition, the staining intensity of the hydrogel+cell group was better than that of the cell group. The histological scores of all other groups were significantly higher than that of the control group at 8 weeks after injection, but the histological score of the hydrogel+cell group was lower than that of the cell group (Figure 9(c)). Except for the hydrogel+cell group and control group, the histological score of the cell group was better than those of the other groups.

As shown in Figure 9(a), the shape of AF was regular and the NP tissue was abundant and rich in water in the control group at 8 weeks after injection. In the PBS group and hydrogel group, the structure of AF was disordered and the area of the NP was significantly reduced and replaced by fibrous tissue. Although the AF tissue was significantly thicker and the area of the NP was significantly reduced, the boundary between the AF and NP was clearer in the hydrogel+cell group and cell group than in the PBS group and hydrogel group. As shown in Figure 9(b), the relative areas of the NP tissue in the hydrogel+cell group and cell group were significantly larger than those of the PBS group and hydrogel group. Furthermore, the relative area of the NP tissue in the hydrogel+cell group was larger than those in the other groups except the control group.

### 3.7. The Cell Apoptosis Analysis

**Figure 10:**
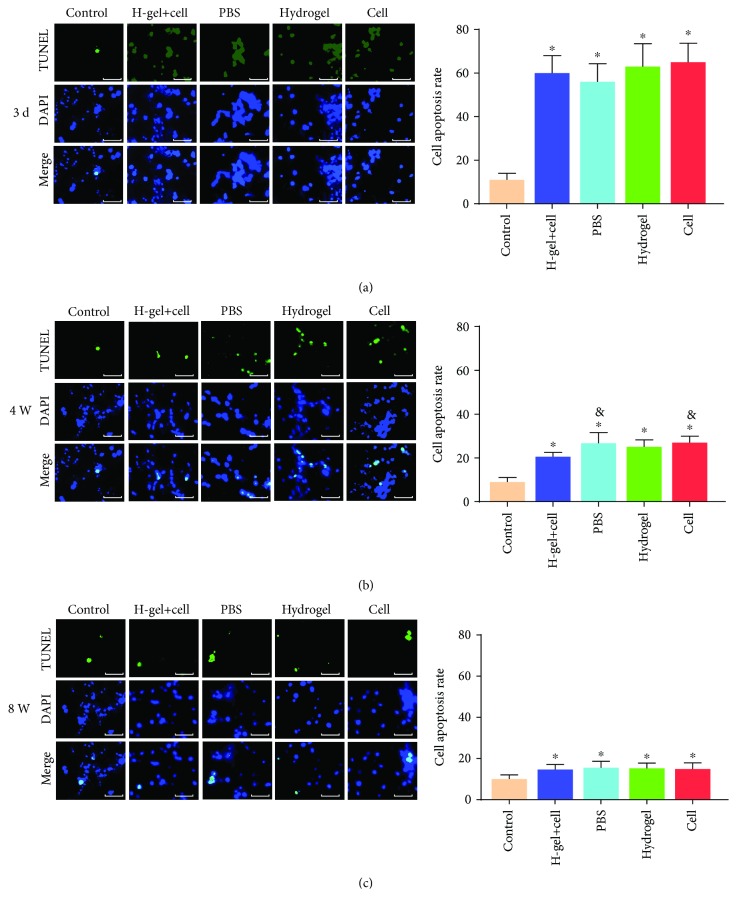
TUNEL staining of different groups. (a) Representative images and the percentage of TUNEL-positive cells in NP tissues at 3 days after operation. (b) Representative images and the percentage of TUNEL-positive cells in NP tissues at 4 weeks after injection. (c) Representative images and the percentage of TUNEL-positive cells in NP tissues at 8 weeks after injection. The H-gel+cell group is the hydrogel+cell group. Data represent mean ± SD; the error bars represent the standard deviation of measurements for 6 separate samples (*n* = 6); ^∗^*p* < 0.05 compared with control group; ^&^*p* < 0.05 compared with hydrogel+cell group. Scale bar = 200 *μ*m.

To elucidate the effect of needle puncture molding and hydrogel in a rat tail disc, we performed TUNEL staining for NP cells and NPMSC. At 3 days after operation, the rate of TUNEL-positive cells in other groups was significantly higher than that of the control group (Figure 10(a)). At 4 weeks after injection, the rate of TUNEL-positive cells decreased significantly and the TUNEL-positive cell rates in the hydrogel+cell group and cell group were lower than those of the hydrogel group and PBS group (Figure 10(b)). With the regeneration of NP tissue and the formation of scar tissue, the rate of TUNEL-positive cells in all groups was significantly lower than those before and there was no significant difference among the groups at 8 weeks after intervention (Figure 10(c)).

### 3.8. Immunofluorescence and RT-PCR Analysis

**Figure 11:**
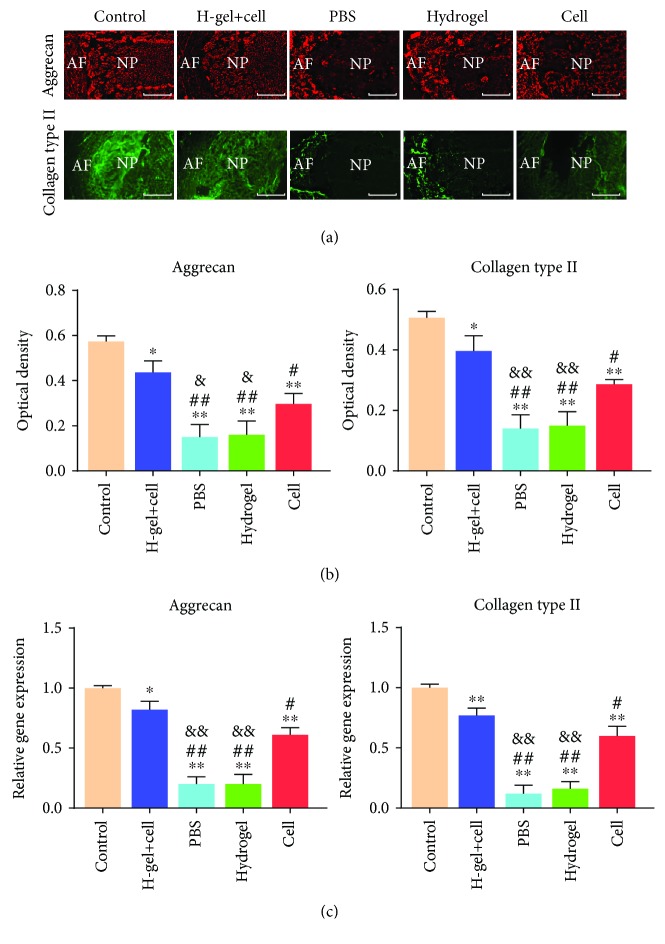
Immunofluorescence and RT-PCR of different groups. (a) Immunofluorescence detection of collagen type II and aggrecan of different groups at 8 weeks after injection. (b) The quantitative analysis of immunofluorescence staining. (c) Gene expression of collagen type II and aggrecan of different groups at 8 weeks after injection. Data represent mean ± SD; the error bars represent the standard deviation of measurements for 3 separate samples (*n* = 3); ^∗^*p* < 0.05, ^∗∗^*p* < 0.01 compared with control group; ^#^*p* < 0.05, ^##^*p* < 0.01 compared with hydrogel+cell group; ^&^*p* < 0.05, ^&&^*p* < 0.01 compared with cell group.

The protein and mRNA expressions of collagen type II and aggrecan were detected at 8 weeks after injection. There were expressions of collagen type II and aggrecan in the control group, hydrogel+cell group, and cell group (Figure 11(a)). Since the normal structure of NP disappeared in the PBS group and hydrogel group, only the smallest area of the NP tissue was found to be positive for collagen type II and aggrecan (Figure 11(a)). As shown in Figures 11(b) and 11(c), the quantitative analysis was significantly higher in both collagen type II and aggrecan in the hydrogel+cell group and cell group than in those in the PBS group and hydrogel group, but significantly lower than those in the control group. The quantitative analysis was higher in collagen type II and aggrecan in the hydrogel+cell group than in those in the other groups except the control group.

## 4. Discussion

Currently, conservation and surgical treatments are often used to treat IDD, but those methods can only relieve symptoms and may even aggravate the possibility of adjacent IDD [6, 29, 30]. Cell transplantation-based biological therapy may be more beneficial to repair and restore the biomechanical function of IVD. Although implanted, autologous NP cells could remain viable, produce ECM, and retain disc height in the degenerative human IVD in an ex vivo study [31]. However, several studies showed that the NP cell lost its ability to differentiate and synthesize ECM [32] and thus has limited application.

MSC have the ability to self-renew and multilineage differentiation; some types of MSC can differentiate into nucleopulpocytes (NPCytes) and produce ECM in the degenerated IVD [10–13]. So far, a variety of MSC have been used [10–13, 33, 34], but the harsh microenvironment of the degenerative IVD [35–37] result in reduced cell viability and ECM synthesis of transplanted MSC [38]. Recently, NPMSC was isolated from normal and degenerate NP tissue and displayed preferable proliferation and better ability to adapt to the harsh IVD microenvironment [39, 40]. Hence, NPMSC may have considerable potential for regenerating IDD. In the present study, the cells isolated from NP had the following characteristics: (1) presenting with spindle-shaped adherent growth and growing in colony formation, (2) higher expressions of CD73, CD90, and CD105 (>95%) and lower expressions of CD34 and CD45 (<5%), (3) higher expressions (>80%) of the NP-progenitor cell surface marker Tie2, and (4) having multilineage differentiation potential. These characteristics met the criteria stated by the ISCT for MSC. Combined with the isolated methods [41, 42], immunophenotypic characterization [9], and cellular morphology [43], the isolated cells were NPMSC indeed.

Both in vitro and in vivo animal studies have demonstrated that MSC exert its regenerative effect in the following ways: (1) differentiating into NPCytes, (2) activating endogenous cells, and (3) producing an anti-inflammatory effect [44–46]. In the present study, X-ray, MRI, and macroscopic observation results demonstrated the regenerative effectiveness of the transplanted NPMSC. The histological staining results also confirmed that IVD with transplanted NPMSC present more regular IVD structure and more ECM. The results of immunofluorescence and RT-PCR further confirmed that the expressions of collagen type II and aggrecan in the NPMSC-transplanted groups (hydrogel+cell group and cell group) were significantly higher than those in the groups without NPMSC (PBS group and hydrogel group). In addition, the transplanted NPMSC survived in the transplanted IVD for at least 30 days visualized by the IVIS system. Those results indicated that NPMSC can survive and produce regenerative effect in the transplanted degenerative IVD.

NPMSC have the potential to differentiate into NPCytes [34], but the harsh microenvironment of the degenerated IVD present a major challenge to insure the survival and function of the implanted cells. Some studies even showed that MSC injected without scaffolds had no regeneration effect [47]. Injecting NPMSC into the IVD tissue has demonstrated its ability to regenerate the degenerative IVD at some degree in the previous and present studies [34]. However, significant obstacles to successful cell therapy still remain, such as leakage of MSC into IVD surroundings, osteophyte formation, and no improvement of IVD height [48]. Therefore, a preferred option may be to encapsulate MSC into a carrier to protect cell leakage and provide a suitable environment to support MSC expansion and differentiation [49]. For this reason, hydrogels are considered an ideal and popular choice to load cells.

Although several studies have focused on the study of IVD cells and MSC transplantation for the treatment of IDD [10, 13, 34], this study was the first to combine allogeneic NPMSC and injectable hydrogel for the treatment of IDD. Several studies have shown that a scaffold with low stiffness is more beneficial for NP-like differentiation [50]. In this study, a polysaccharide hydrogel modified with 3D RGD peptide was used, which can mimic the condition of the natural ECM of NP and provide binding sites for cells. The RGD peptide creates cell-adhesive structures in the hydrogel and greatly increases the long-term cell viability in 3D cultures [51]. The result of in vitro study showed that the hydrogel concentration of 1 : 2 *v*/*v* was most suitable for cell proliferation. This may be attributed to the fact that high crosslinking hydrogel can hinder the nutrient transport, but the low crosslinking hydrogel is less relatively rigid to provide a good scaffold for cells. Some studies found that 3D culture and spherical cell morphology could promote the viability and differentiation of loaded cells [52, 53]. The present study also demonstrated that the hydrogel can promote NPMSC proliferation and induce it to differentiate in vitro confirmed by the results of cell counting and quantified analysis of collagen type II and aggrecan. The result of cell tracking further indicated that the luminescence of ROI in the hydrogel+cell group was significantly stronger than that in the cell group, and the rate of TUNEL-positive cells in the hydrogel+cell group was lower than that in the cell group. All those results indicated that hydrogel used as a carrier in the present study can support the long-term survival of NPMSC in vivo and induce NPMSC to differentiate into NP cells.

The amounts of ECM and hydration state of the NP are of great importance to the function of normal IVD. There were increased expressions of collagen type II and aggrecan in the hydrogel+cell group compared with the cell group. MRI index can be considered an indirect indicator to reflect the hydration state of NP tissue. There was also a significantly higher MRI index in the hydrogel+cell group compared with the cell group. However, the hydrogel alone did not regenerate the NP tissue as illustrated in the hydrogel group. Those results indicated that hydrogel can promote the regenerative effect of the transplanted NPMSC to enhance ECM synthesis, and the hydrogel used in the present study can increase the effect of the transplanted NPMSC on the hydration of degenerative IVD. In addition, being preliminary, this study still has some limitations. First, the use of a rat model of IDD cannot fully reflect the natural course of human IDD; it may be more representative to use large animals as models. Second, there is no related research on the mechanical properties of the hydrogel, which is critical for the dispersion of the IVD stress. Third, short experimental duration of this study is insufficient to reflect the complete effect of the hydrogel-loaded NPMSC transplantation on the repair of degenerative IVD; future studies should extend the experiment duration to a longer time.

## 5. Conclusion

This study demonstrated that the hydrogel modified with 3D RGD peptide could promote the proliferation and differentiation of NPMSC in vitro. Moreover, the hydrogel could promote NPMSC's long-term retention and survival in the degenerative IVD and ECM secretion. NPMSC-alone transplantation could repair degenerative IVD to a certain extent, but the hydrogel-loaded NPMSC offered a better effective and promising therapeutic strategy for IVD regeneration. Therefore, hydrogel combined with NPMSC has a potential therapeutic value in the regeneration of IVD.

## Figures and Tables

**Table 1:** Primers used for reverse transcription quantitative polymerase chain reaction.

Gene	Primer/probe sequence
Collagen type II	5′-CATCCCACCCTCTCACAGTT-3′
	5′-ACCAGTTAGTTTCCTGCCTCTG-3′

Aggrecan	5′-TCCACAAGGGAGAGAGGGTA-3′
	5′-GTAGGTGGTGGCTAGGACGA-3′

*β*-Actin	5′-GGACTTCGAGCAAGAGATGG-3′
	5′-GATGGAGTTGAAGGTAGTTTCG-3′

## Data Availability

The data used to support the findings of this study are available from the corresponding authors upon request.
